# pH Sensing Properties of Co_3_O_4_-RuO_2_-Based Electrodes and Their Application in Baltic Sea Water Quality Monitoring

**DOI:** 10.3390/s25041065

**Published:** 2025-02-11

**Authors:** Kiranmai Uppuluri, Dorota Szwagierczak, Krzysztof Zaraska, Piotr Zachariasz, Marcin Stokowski, Beata Synkiewicz-Musialska, Paweł Krzyściak

**Affiliations:** 1 Łukasiewicz Research Network—Institute of Microelectronics and Photonics, Kraków Division, ul. Zabłocie 39, 30-701 Kraków, Poland; dorota.szwagierczak@imif.lukasiewicz.gov.pl (D.S.); krzysztof.zaraska@imif.lukasiewicz.gov.pl (K.Z.); piotr.zachariasz@imif.lukasiewicz.gov.pl (P.Z.); beata.synkiewicz.musialska@imif.lukasiewicz.gov.pl (B.S.-M.); 2 Institute of Oceanology of the Polish Academy of Sciences (IOPAN), ul. Powstańców Warszawy 55, 81-712 Sopot, Poland; stokowski@iopan.pl; 3 Department of Infection Control and Mycology, Faculty of Medicine, Jagiellonian University Medical College, Czysta 18, 31-121 Kraków, Poland; pawel.krzysciak@uj.edu.pl

**Keywords:** cobalt oxide, ruthenium oxide, pH electrode, screen printing, potentiometric sensors, water quality monitoring, the Baltic Sea

## Abstract

Water is critical for the sustenance of life and pH is an important parameter in monitoring its quality. Solid-state pH sensors provide a worthy alternative to glass-based electrodes due to many advantages such as low cost, longer shelf life, simpler manufacturing, easier operation, miniaturization, and integration into electronic systems. Cobalt oxides are relatively cheaper and more abundantly available than ruthenium oxide. This work aims to reduce the environmental impact of screen-printed pH sensors by mixing Co_3_O_4_ and RuO_2_ in five molar proportions (30%, 40%, 50%, 60%, and 70%) and investigating the influence of oxide proportions on the pH-sensing properties of the resulting composition using potentiometric characterization, scanning electron microscopy, X-ray diffraction, surface profilometry, and electron dispersive spectroscopy. Although all the developed compositions showed super- or near-Nernstian sensitivity with good linearity, the sensors based on 50 mol% Co_3_O_4_-50 mol% RuO_2_ were the best due to superior sensitivity, selectivity, and stability. Fabricated sensors were applied in real-life environmental, municipal, and commercial water samples, including those from various depths in the Baltic Sea, and were found to be accurate in comparison to a glass electrode.

## 1. Introduction

Water quality monitoring is an essential tool for effective public and ecological health protection. The various parameters related to water quality may be chemical, biological, or physical, and their measurement is useful to indicate whether the water is polluted or not. Sensor technology and communications are at the forefront of quality control of water. The measurement of pH is critical for water quality monitoring because it influences many chemical and biological processes in the environment [1]. For this reason, standards for water quality include guidelines for pH by key organizations such as the World Health Organization (WHO), the United States Environmental Protection Agency (EPA), and the European Union (EU).

Electrochemical pH sensors may be potentiometric, resistive, capacitive, or conductimetric in principle [2]. The traditional method of pH measurement relies on the potentiometric principle, and it is based on a glass electrode [3]. However, the glass electrode is difficult to fabricate due to its complex structure. The presence of liquid in its construction and a glass body make the electrode fragile and easily prone to breakage in turbulent waters and harsh environments. The potential for miniaturization is severely restricted due to the presence of an electrolyte solution in the electrode body and the absence of flexibility [2]. This makes it very challenging to integrate a glass electrode into modern electronic systems which tend to avoid bulky designs. In order to assist the growing demand and preference for green and transient electronics, modern sensor systems need to adopt materials and designs that align with objectives of the current sustainability-oriented times. Thick-film pH sensors offer a worthy alternative to the glass electrode due to their low cost, simple fabrication, small size, and easy operation. To fabricate thick-film electrodes, screen-printing technology is suitable because this inexpensive and simple method allows flexibility in design and mass production [2].

In 1989, Fog and Buck were the first to find that oxides of platinum group metals (PGM), such as ruthenium (IV) oxide (RuO_2_) and iridium (IV) oxide (IrO_2_), exhibited sensitivity to changes in pH [4]. Less expensive in comparison to a commercial glass electrode, the majority of the oxides of PGMs are electronically conducting materials and mimic the proton-exchange processes that take place on the pH-sensitive bulb of a glass pH electrode, thereby earning the title of ion-exchange surfaces [5]. Moreover, the good pH-sensing properties of RuO_2_, such as near-Nernstian sensitivity, low drift rate, fast response, long-term stability, good selectivity, the ability to maintain the potentiometric characteristics in a wide range of pH and temperature, as well as their successful application for the pH measurement of real-life water and food samples, were previously confirmed [6,7,8]. Nevertheless, PGMs are still rare in the Earth’s crust, expensive and have a significant environmental impact throughout their entire life cycle from cradle to grave; substitution is suggested as one of the innovative ways to reduce their content in the final products [9]. Researchers attempted to reduce its content in sensing layers by replacing or combining it with other less expensive and more abundant metal oxides, such as SnO_2_ [10], TiO_2_ [11], Ta_2_O_5_ [12], La_2_O_3_ [13], CuO [14], Cu_2_O [15], Co_2_O_3_ [16]_,_ ZnO [17]_,_ WO_3_ [18], and Sm_2_O_3_ [19]. Advancements in metal oxides-based pH sensors destined for various chemical, biological, environmental, food, and medical applications have been recently described in several review papers [2,20,21,22,23]. However, it is not clear how the sensing behavior of the mixed compositions changed in relation to the proportions of starting oxides used during development of the pH-sensing layers. Knowledge of this relationship is helpful in choosing the most appropriate proportions based on specific requirements such as operational conditions and final applications. A study investigating the pH-sensing properties of cobalt oxide mixed with graphite ink in the ratio range of 2–15% found that the mixture containing 10% of cobalt oxide yielded the highest sensitivity (60 mV/pH), and the best correlation coefficient [24].

Metal oxides such as copper oxide and cobalt oxide are comparatively more abundant and cheaper but on their own, they do not perform as well as RuO_2_ [24,25,26]. In case of cobalt which is the 32nd most abundant element in our planet’s crust, this may be due to the relatively low electrocatalytic activity and poor conductivity of cobalt [27]. On the other hand, the ability of cobalt oxide to provide a higher number of OH sites on the material/solution interface of the electrode due to large surface areas of fine grains [24] could positively contribute to the pH-sensing mechanism on the surface of a screen-printed electrode based on cobalt oxide and ruthenium oxide. Cobalt oxide also has intrinsically high activities at pH close to 7 [27], which resembles the range of pH value for most types of water.

This work attempts to comprehensively study the impact of molar proportions on the sensing properties and overall sensor behavior of a mixed composition made from ruthenium oxide RuO_2_ and cobalt (II, III) oxide Co_3_O_4_. In addition to the potentiometric characterization of properties such as sensitivity, stability, selectivity, response time, and drift rate, advanced analytical methods such as scanning electron microscopy and X-ray diffraction have been used to analyze the microstructural and compositional properties of the screen-printed layers. Finally, fabricated electrodes were used to measure the pH of real-life water samples from the tap, mineral water bottle, river, lake, and various depths of the Baltic Sea.

## 2. Materials and Methods

### 2.1. Fabrication of Sensors

All the thick-film pastes for pH-sensitive layers were prepared in the laboratory using high-purity starting metal oxide powders (>97%, Sigma-Aldrich, St. Louis, MO, USA). After calculating the required amount of each metal oxide based on the desired proportion in the combined composition, the metal oxides were first weighed, and then, the mixture was put into a planetary ball mill (Pulverisette 5, Fritsch GmbH, Idar-Oberstein, Germany) and wet-milled in isopropyl alcohol with zirconia balls for 8 h. After the removal of the isopropyl alcohol during drying, a fine powder of a homogenous mixture of the metal oxides was obtained which was ready to be converted into a paste. To prepare the pastes for sensing electrodes, the oxide powder was mixed with a binder (ethyl cellulose) and solvent (anhydrous terpineol) in an agate mortar. The three components were then grinded for about 20 min until the rheology of the resultant paste was optimal for the screen-printing process. The optimal rheology of the paste should be adjusted to the mesh size of the screen used to print the electrodes. Sensitive layers for pH sensors in this research were screen-printed using a 150-mesh screen.

**Figure 1:**
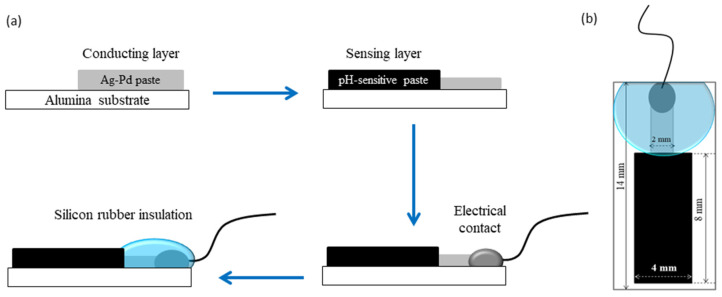
(**a**) Flow diagram of the fabrication process; (**b**) dimensions of the Co_3_O_4_-RuO_2_-based pH electrode.

For the fabrication of the pH-sensitive electrodes, firstly a conducting layer of the Ag/Pd paste (9695, Electro-Science Laboratories, King of Prussia, PA, USA) was screen-printed on an Al_2_O_3_ (96%) substrate. The conducting layer was allowed to dry for 15 min at 120 °C in a dryer before being fired at 860 °C for 15 min. After firing of the conductive layer, the paste based on Co_3_O_4_-RuO_2_ was screen-printed at one end of the conductive layer, dried at 120 °C for 15 min and sintered at 900 °C for one hour. For comparison, pastes based on three pure cobalt oxides—CoO, Co_3_O_4_, and Co_2_O_3_—were prepared and used for fabrication of pH sensors following the same fabrication procedure used for the electrodes based on Co_3_O_4_ and RuO_2_. After sintering of the sensitive layer, a copper wire was soldered at the other end of the conducting Ag/Pd layer. This electrical contact between the wire and the layer was insulated using a non-corrosive polydimethylsiloxane (DOWSILTM 3140 RTV coating, Midland, TX, USA) coating while the sensitive area of the electrode was left uncovered. The schematic representation of the steps involved in the fabrication of the pH-sensing electrode and its dimensions are shown in Figure 1.

### 2.2. Morphological, Compositional, and Structural Analysis

The phase compositions of the various pH-sensitive thick films were examined by the X-ray diffraction method (XRD) (D8 Advance Eco, Bruker, Billerica, MA, USA). The microstructure, elemental composition, and compatibility with the alumina substrate of the sintered pH electrodes were studied using scanning electron microscopy (SEM) and X-ray energy-dispersive spectroscopy (EDS) (Quattro S Thermo Fisher Scientific, Loughborough, UK). Measurements of the thickness and roughness of the fired screen-printed sensing electrodes were performed in the cooperation with Technolutions company (Łowicz, Poland) using an optical profilometer (UP-3000, Rtec Instrument Inc., San Jose, CA, USA).

### 2.3. pH Measurement

The *emf* was measured using a multimeter (6.5-digit Series 2002, Keithley Instruments, Cleveland, OH, USA) which was connected to a computer, and data were analyzed using LabVIEW program (National Instruments, Austin, TX, USA). The reference electrode was an Ag/AgCl/KCl glass electrode (HYDROMET, Gliwice, Poland), and a commercial pH meter (ELMETRON, Zabrze, Poland) was used for cross-checking the pH of the test solution. Buffer solutions were purchased from Chempur (Piekary Śląskie, Poland).

After measuring the *emf* value in each buffer solution ranging from 2 to 12, the sensitive and reference electrodes were washed with deionized water and dried gently with a paper towel. Electrode sensitivity, *E*^0^, and linearity of the response were determined by plotting the electrode potential as a function of pH and obtaining the linear equation describing the relationship between them. Electrode sensitivity was calculated as the slope of the linear equation, *E*^0^ was calculated as the potential at pH = 0 by extrapolating the data, and the linearity of the response of the electrode to pH change was calculated as the correlation coefficient (R^2^). The response time was determined as the time needed for the electrode potential to reach 90% of the stable value.

To observe the stability of response and calculate the drift rate during long and continuous measurements, the sensors were submerged in pH 1, pH 4, pH 7, pH 10, and pH 13 buffers for 24 h, and the drift over time in the *emf* (mV) was calculated per hour. For measuring the selectivity of the sensitive electrode, the effect of interfering ions on the sensing performance of the fabricated pH electrodes was investigated by calculating the sensitivity in pH buffers containing 0.01M concentration of KCl, CaCl_2_, MgCl_2_, Na_2_SO_3_, and NH_4_NO_3_. KCl, CaCl_2_, and Na_2_SO_3_ was purchased from Chempur (Piekary Śląskie, Poland), MgCl_2_ was purchased from Stan Lab (Lublin, Poland), and NH_4_NO_3_ was purchased from Sigma Aldrich (St. Louis, MO, USA). The influence of temperature on the performance of the fabricated electrodes was investigated by monitoring their *emf* responses in pH 4, 7, and 10 buffer solutions. The pH-sensing electrodes were immersed in semi-frozen buffers, which were then heated gradually to 60 °C using a laboratory hot plate (MS 11 H, WIGO, Pruszków, Poland). The *emf* response was continuously recorded in real time throughout the entire duration as the temperature increased from 0 to 60 °C in order to assess the stability of the pH electrodes in the pH range of 4–7, as well as the tolerance of the screen-printed pH-sensing layer to variations in temperature.

### 2.4. Application in Water Samples

**Figure 2:**
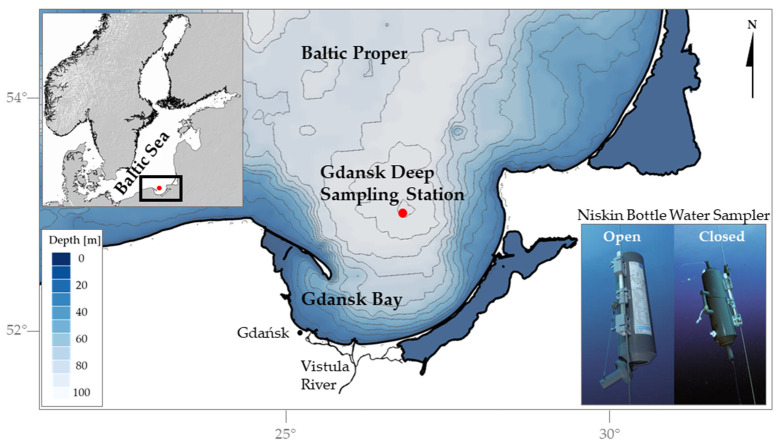
The location of the sampling station and the sampling method employed in the study (after General Oceanics Inc.). The upper left corner contains an inset showing a broader map of the region for geographic context. The central section of the figure highlights the study area, zooming in on the Gdańsk Deep sampling station. The lower right corner illustrates the Niskin Bottle Water Sampler in two positions: open and closed.

Fabricated electrodes were investigated for their accuracy in comparison with a glass electrode in water samples from the tap, commercial mineral water bottle (still water, Wysowianka Zdrój), Zakrzówek Lake, and Vistula River in Kraków, Poland, as well as from five depths (0 m, 2 m, 42 m, 72 m, and 100 m) of the water column of the Baltic Sea. Marine water samples were collected during spring 2024 from the deepest part of the Gdańsk Deep, situated in the southern Baltic Sea (54°46.231′ N, 19°11.655′ E) as indicated in Figure 2.

Collection was carried out aboard the research vessel (RV) Oceania using a Niskin bottle water sampler by General Oceanics Inc. (Miami, FL, USA) (Figure 2) in a manner that prevented gas exchange and atmospheric air contamination. The samples were transferred from the Niskin bottle to PET bottles which were pre-cleaned with 10% hydrochloric acid and distilled water. To minimize the risk of contamination during the transferring process, the PET bottles were thoroughly rinsed three times with the water being sampled. Then, they were tightly sealed and stored in a refrigerator at 4 °C until the measurement.

### 2.5. Anti-Bacterial Properties of the Electrode

As described in Section 2.1, the pH electrode contains a screen-printed layer of a conductive Ag/Pd paste and a pH-sensitive RuO_2_-Co_3_O_4_ paste. In this preliminary test, a pH-sensing layer containing 50 mol% RuO_2_ and 50 mol% Co_3_O_4_ was placed at the bottom of a Petri dish. It was then covered with Mueller Hinton Agar, which was swabbed with a bacteria standard solution (*E. coli*, DSM 5695) at a concentration of 106 CFU/mL. The samples underwent incubation for 20 h at 35 °C. Post-incubation, a visual inspection was conducted to assess the inhibition of bacterial growth above and around the sample.

## 3. Results and Discussion

### 3.1. Phase Composition and Morphological Analysis

**Figure 3:**
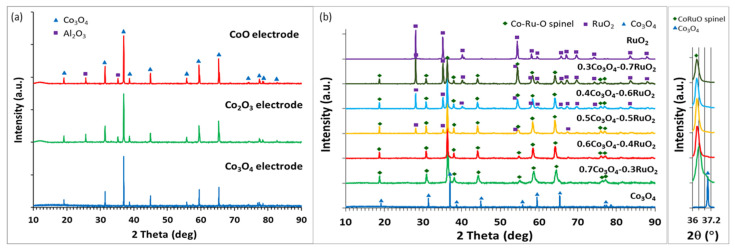
X-ray diffractograms of screen-printed layers of the various cobalt oxides (**a**) and the various compositions based on Co_3_O_4_ and RuO_2_ in different molar proportions (**b**).

The XRD analysis of thick films based on pure cobalt oxides (CoO, Co_3_O_4_, and Co_2_O_3_) revealed that the electrodes fired at 900 °C exhibited peaks attributed to one cobalt oxide phase—Co_3_O_4_, independently of the composition of starting cobalt oxides, as shown in Figure 3a. In addition, some peaks originating from the Al_2_O_3_ substrate were found. In the temperature range of 300–900 °C, Co_3_O_4_ was the most thermodynamically stable cobalt oxide (above 940 °C, Co_3_O_4_ decomposes and the CoO phase is expected to be formed, but such high firing temperatures were not used in this work).

Figure 3b shows the XRD patterns for xCo_3_O_4_-(1-x)RuO_2_ thick-film electrodes for various molar ratios of the component oxides (x = 0, 0.3, 0.4, 0.6, and 0.7). The analysis revealed three crystalline phases—nonstoichiometric ruthenium cobalt spinel oxide Co_2.35_Ru_0.65_O_3.86_ which crystallized in the cubic space group I-42d (ref. code 98-016-2402), ruthenium (IV) oxide RuO_2_ which crystallized in the tetragonal space group P42/mnm (ref. code: 98-001-5071), and cobalt (II, III) oxide Co_3_O_4_ which crystallized in the cubic space group Fd-3m (ref. code 98-006-9366). For xCo_3_O_4_-(1-x)RuO_2_ compositions containing 30, 40, and 50 mol% Co_3_O_4_ in the starting mixture, after sintering at 900 °C, the ruthenium cobalt spinel phase was a dominant crystalline phase with an addition of the RuO_2_ phase. XRD analysis after the sintering of the electrodes containing 60 and 70 mol% Co_3_O_4_ in unfired state revealed a single-phase composition based on the Co_2.35_Ru_0.65_O_3.86_ spinel. Figure 3b distinctly illustrates the diminishing content of the RuO_2_ phase in the sintered films with an increasing content of Co_3_O_4_ in the starting mixtures and the complete incorporation of Ru ions into the spinel crystal structure for 0.6Co_3_O_4_-0.4RuO_2_ and 0.7Co_3_O_4_-0.3RuO_2_. The inset in Figure 3b shows that the position of the main peak (100% intensity) related to Co_2.35_Ru_0.65_O_3.86_ remained unchanged for the compositions containing 30, 40, 50, and 60 mol% Co_3_O_4_. However, for the 0.7Co_3_O_4_-0.3RuO_2_ composition (with the highest Co_3_O_4_ content in the starting composition), this peak was slightly shifted towards higher 2 theta values. This effect is related to decreasing lattice parameters of the spinel due to the smaller ionic radius of a cobalt ion as compared with a ruthenium ion. Similarly, the two major phases in a RuO_2_-based pH-sensing electrode doped with 20 mol% Cu_2_O were RuO_2_ and Cu-Ru-O complex oxide [15]. On the other hand, no additional phase was found to be formed due to the reaction of two component oxides when RuO_2_ was mixed with TiO_2_ (70:30 mol%) [2], Ta_2_O_5_ (70:30 mol%) [2], SnO_2_ (73:27 mol%) [10], and CuO (50:50 mol%) [14].

**Figure 4:**
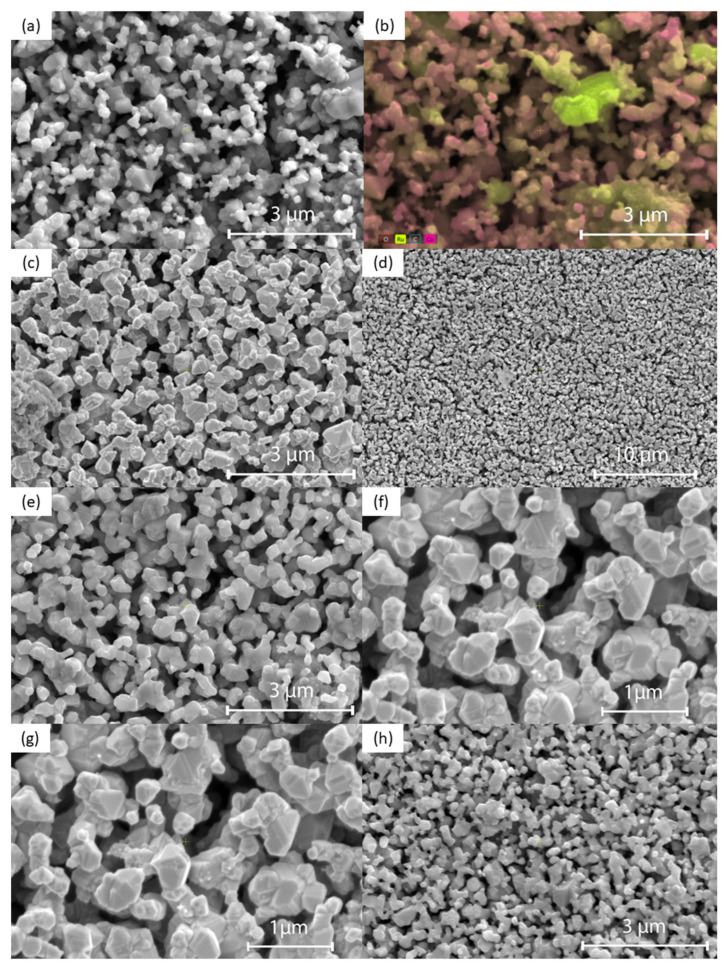
SEM images of the surface of Co_3_O_4_-RuO_2_ thick-film electrodes with different Co_3_O_4_ contents: (**a**) 30 mol% Co_3_O_4_ (50,000×, 10 kV); (**b**) 30 mol% Co_3_O_4_ (50,000×, 15 kV; SEM with EDS analysis to show Co and Ru distribution with the yellow regions corresponding to RuO_2_ grains); (**c**) 40 mol% Co_3_O_4_ (50,000×, 3 kV); (**d**) 40 mol% Co_3_O_4_ (12,000×, 10 kV); (**e**) 50 mol% Co_3_O_4_ (50,000×, 10 kV); (**f**) 50 mol% Co_3_O_4_ (100,000×, 3 kV); (**g**) 60 mol% Co_3_O_4_ (50,000×, 10 kV); (**h**) 70 mol% Co_3_O_4_ (50,000×, 10 kV).

**Figure 5:**
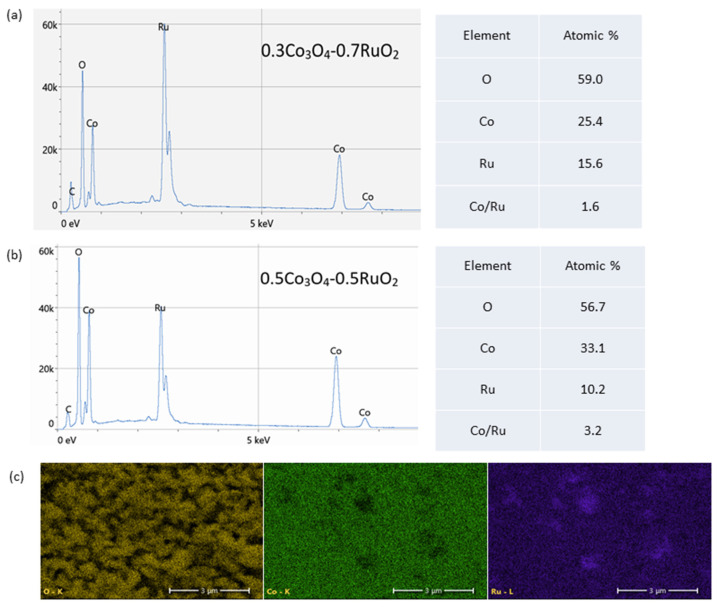
Results of EDS analysis: (**a**) EDS spectra and elemental composition of 0.3Co_3_O_4_-0.7RuO_2_; (**b**) EDS spectra and elemental composition of 0.5Co_3_O_4_-0.5RuO_2_; (**c**) EDS maps of O (yellow), Co (green), and Ru (purple) for 0.5Co_3_O_4_-0.5RuO_2_.

Figure 4 shows the SEM images of the surface morphology of Co_3_O_4_-RuO_2_ thick-film electrodes containing 30, 40, 50, 60, and 70 mol% Co_3_O_4_, screen-printed on an Al_2_O_3_ substrate and fired at 900 °C. The morphology of the electrodes was porous and fine-grained. The grain sizes were not uniform, changing from 200 nm to 1.5 μm. The presence of two crystalline phases, cobalt ruthenium spinel as a major phase and RuO_2_ as a minor phase, should be expected for the electrodes containing 30, 40, and 50 mol% Co_3_O_4_ based on the XRD results. This effect is illustrated in Figure 4b which presents the SEM image along with the result of EDS analysis for the electrode containing 30 mol% Co_3_O_4_. Some of the bigger grains can be assigned to RuO_2_ (the yellow color indicates Ru), while the majority of grains with different grain sizes can be attributed to the Co-Ru spinel phase (the pink color indicates Co). Figure 5a,b illustrate the EDS spectra and elemental compositions of the sensing layers made of 0.3Co_3_O_4_-0.7RuO_2_ and 0.5Co_3_O_4_-0.5RuO_2_ pastes. The sintered layers contained three component elements—Co, Ru, and O, in atomic proportions close to those for the starting compositions (a small detected amount of C originated from surface contamination). The EDS analysis showed that the Co/Ru atomic ratio was 1.6 for 0.3Co_3_O_4_-0.7RuO_2_ (the ratio calculated based on the starting composition was 1.3) and 3.2 for 0.5Co_3_O_4_-0.5RuO_2_ (the ratio calculated based on the starting composition was 3). The results of EDS mapping of O, Co, and Ru for 0.5Co_3_O_4_-0.5RuO_2_, shown in Figure 4c, illustrated the uniform distribution of all elements and bigger sizes of RuO_2_ grains as compared with spinel grains containing both cobalt and ruthenium. However, it should be taken into account that due to very fine grains with submicron sizes, applicability of the EDS analysis to such microstructures of the layers is limited.

Profilometry results revealed the various parameters of the roughness profile for the screen-printed thick-film pH-sensing layers which are listed in Table 1. The analysis using an optical profilometer showed a relatively high surface roughness (R_a_ = 1.1 µm) of the thick-film electrodes which according to the site-binding model is expected to be advantageous for the detection sensitivity due to an increase in the total number of surface sites [19]. Potentiometric characteristics of screen-printed thick-film electrodes are also influenced by the thickness of the sensing layer in terms of response time whereby it increases as the thickness of the film increases [24]. The average thickness recorded for the electrodes fabricated in this study was about 11 µm. Based on their requirements, researchers and manufacturers can regulate the thickness of their electrodes by using screen-printing technology to fabricate them.

### 3.2. Potentiometric Characteristics

pH sensitivity of the electrodes can be calculated as the slope of the Nernstian relation between the electromotive force (*emf*) of the cell and pH. An electrical characteristic of the electrochemical cell, *emf*, is the difference between the potentials recorded at the sensitive electrode and reference electrode (*E*). The Nernst Equation (1) describes the half-reaction taking place at the sensitive electrode in terms of *E*^0^, the standard potential (V); *R*, the universal gas constant (8.314 J/K.mol); *T*, temperature (K); *F*, Faraday’s constant (96,485 C/mol); *n*, the number of electrons participating in the redox reaction; and [*Red*] and [*Ox*], activities of the reduced and oxidized forms of the sensing electrode material (mol/L):

(1) $$E=E^{0}-\frac{RT}{nF}ln\frac{\left[Red\right]}{\left[Ox\right]}$$

Prior to combining RuO_2_ and Co_3_O_4_, their pH-sensing properties were tested individually. Pure RuO_2_ and Co_3_O_4_ were screen-printed based on the exact same fabrication process. In comparison to Co_3_O_4_, RuO_2_ exhibits far superior pH-sensing characteristics such as near-Nernstian sensitivity (61.8 ± 1.0 mV/pH), fast response, a wide range of pH, tolerance to high temperatures, and good stability, repeatability, and reversibility [6]. In contrast, screen-printed Co_3_O_4_-based pH sensors are unstable (poor repeatability, reversibility, and drift rate) and have lower pH sensitivity (42.8 ± 16.8 mV/pH) (Table 2). Therefore, the major disadvantages of using RuO_2_ are the high material cost and low availability, whereas the major disadvantage of using Co_3_O_4_ is the poor pH-sensing performance. Both materials compensate for each other’s disadvantages with their own advantages. Due to this reason, they were chosen as the starting powders in this study which aims to propose a cost-effective, environmentally friendly, sensitive, and reliable pH-sensing layer.

**Figure 6:**
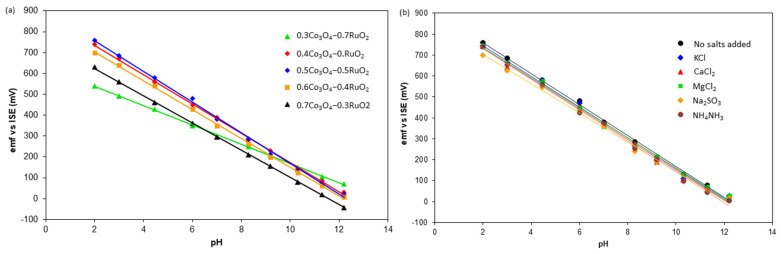
The *emf* of sensors versus pH for different electrodes: (**a**) electrodes with different proportions of Co_3_O_4_ and RuO_2_; (**b**) 50 mol% Co_3_O_4_-50 mol% RuO_2_ electrode in the presence of interfering salts.

The pH sensitivity, linear correlation R^2^, and standard cell potential *E*^0^ of Co_3_O_4_-RuO_2_ electrodes with various Co_3_O_4_ contents are presented in Table 2. Figure 6a shows the *emf* of pH sensors as a function of the Co_3_O_4_ content in the sensing electrode. It was found that all the combinations of Co_3_O_4_ and RuO_2_ were sensitive to pH with linearity close to 1. This confirms that the compositions developed using Co_3_O_4_ and RuO_2_ in the range between 30 mol% Co_3_O_4_-70 mol% RuO_2_ and 70 mol% Co_3_O_4_-30 mol% RuO_2_ are empirically validated candidates for pH-sensing applications.

The highest pH sensitivity was exhibited by the pH electrode based on 50 mol% Co_3_O_4_-50 mol% RuO_2_ followed by that based on 40 mol% Co_3_O_4_-60 mol% RuO_2_. The rate of decrease in pH sensitivity after exceeding 50 mol% Co_3_O_4_ in the starting material was less than after receding. Therefore, similar electrochemical properties can be found in the compositions containing more Co_3_O_4_ than RuO_2_ as compared to compositions containing more RuO_2_ than Co_3_O_4_. These results are a positive indication that the content of PGMs in the sensing layer of screen-printed pH sensors may be reduced without a tradeoff with performance. Successful reduction in the RuO_2_ content in pH electrodes based on mixed oxides has been also attained by other authors [2,10,11,13,14,15]. However, super-Nernstian behavior, which implies that the pH sensitivity of the fabricated electrodes exceeds the theoretical pH sensitivity of 59.1 mV/pH, was not obtained in those compositions. For example, when 70 mol% RuO_2_ was mixed with 30 mol% TiO_2_ using the Pechini method on a Ti substrate, the pH sensitivity of 56mV/pH was achieved [11]. A similar pH sensitivity was observed when 73 mol% RuO_2_ was mixed with 27 mol% SnO_2_ [10]. On the other hand, a super-Nernstian pH response (65 mV/pH) was obtained for a sol-gel derived RuO_x_-based pH sensor used for the assessment of glucose [28].

pH-sensing layers based on 30, 40, and 50 mol% Co_3_O_4_ in the starting powder had a fast response time which remained below 15 s and a low drift rate of 0.02 mV/h on average, in the pH range of 2–12. Sensing layers based on starting powders with higher than 50 mol% Co_3_O_4_ content tended to have a slower response time which might take up to a minute, a higher drift rate of 0.11 mV/pH, and poor stability of the response signal due to noise.

In this study, RuO_2_ is one of the two major phases detected in the pH-sensing layers containing at least 50 mol% of RuO_2_ in the starting composition. Among the sensing mechanisms of metal oxides proposed by Fog and Buck [4], oxygen intercalation was found to be the most probable [2]. In Equation (1), the potential difference between the reference and the sensitive electrode is proportional to pH, and it is generated as a result of the formation of oxide sites that release protons and cause the metal oxide to be in a state of mixed valency. These oxide sites arise from the dissociative adsorption of water which covers the surface of the sensing layer with hydroxide groups, and the ion exchange process changes the potential difference as the pH of the solution varies [2]. It was also indicated previously that, in the pH-sensing layers based on binary metal oxide compositions with higher RuO_2_ content, mixed electronic and ionic conduction is the dominant mechanism [2]. The other major phase detected in all the pH-sensing layers developed in this study is a non-stoichiometric Co-Ru complex oxide. The oxygen deficiency in the spinel Co-Ru oxide escalates the formation of oxygen vacancies, restricts the kinetic barriers and enhances overall electro-catalytic characteristics by supporting the creation of secondary active surface phases and regulating the charge transfer and bulk electronic properties of the pH-sensing electrode [27].

**Figure 7:**
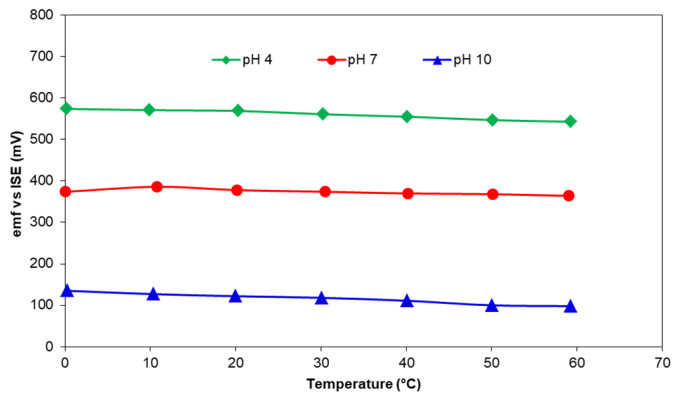
The influence of temperature on the *emf* response of 50 mol% Co_3_O_4_-50 mol% RuO_2_ in various pH buffers.

However, electrochemical properties alone do not determine the quality of a sensor. A screen-printed sensing layer must also be durable and able to withstand harsh physical conditions such as turbulent flow and the presence of large particles in the water. Unlike the nominal and acceptable differences in the potentiometric performance, there is a significant difference between various compositions in terms of their adhesion to the alumina substrate which subsequently impacts a very important feature of the sensor—long-term stability. Under the conditions of synthesis, printing, and sintering of pH-sensing layer, used in this study, the composition in Table 2 that had the best adhesion to the substrates and sustained repeated measurements without peeling off or being washed away was 50 mol% Co_3_O_4_-50 mol% RuO_2_. The sensing layer was able to withstand repeated experiments and storage in dry ambient conditions over a period of 6 months while exhibiting near-Nernstian sensitivity with a good correlation coefficient. When the temperature of the buffers at pH of 4, 7, and 10 was raised from 0 to 60 °C, there was a slight change in the *emf* due to the increased dissociation process which followed the Nernst theory (Equation (1)) and led to higher participation of OH^−^ and H^+^ ions in the electrochemical reactions. The *emf* of the sensing layer based on 50 mol% Co_3_O_4_-50 mol% RuO_2_ adapted instantaneously to the rising heat from 0 to 60 °C (Figure 7) and validated the stability of the fabricated sensors in the presence of continuously varying temperature. A similar result was observed for pH electrodes based on Co_2_O_3_-RuO_2_ pH electrodes [16].

During the experiment investigating the influence of temperature on the *emf* response of the fabricated pH-sensing electrodes, sensors based on 50 mol% Co_3_O_4_-50 mol% RuO_2_ which were fabricated 4 years ago were also tested to investigate the changes in pH-sensing characteristics over time. In comparison to the freshly prepared Co_3_O_4_-RuO_2_-based pH electrodes, they preserved good sensitivity although they had a slower response and took longer to adapt to the continuously changing pH due to the consistently rising temperature of the pH buffer. In the experiment concerning the influence of interfering ions on developed pH electrodes, the old sensors generally maintained the proper response. Their pH sensitivity was notably lower (45.3 ± 4.5 mV/pH) as compared with that of the new sensors but the linearity remained close to 1. The influence of aging on the response time during rising temperature was not observed at stable room temperature. Despite a higher standard deviation among the older sensors (±4.5 mV/pH) compared to that of the new ones (±1.1 mV/pH), their ability to function reliably after four years highlights their reliable long-term stability. In contrast, sensors less than one year old maintained excellent response time and stability over extended periods in a wide range of temperature.

The sensors show repeatable pH-sensing behavior in the pH range of 2–12, as evidenced by the low standard deviation in Table 2. The standard deviation in pH sensitivity for the best composition based on the 50 mol% Co_3_O_4_-50 mol% RuO_2_ was ±1.1 mV/pH, and for all the developed compositions, an average was ±3.7 mV/pH. However, the standard deviation among electrodes was much lower when they were used to measure the pH of water samples as seen in Table 3.

The pH sensitivity of the fabricated electrodes was also investigated in 0.01 M solutions of water mixed with KCl, CaCl_2_, MgCl_2_, Na_2_SO_3_, and NH_4_NO_3_. All the fabricated electrodes continued to exhibit super-Nernstian sensitivity in the presence of the aforementioned salts, as illustrated in Figure 6b. The pH sensitivity of the 50 mol% Co_3_O_4_-50mol% RuO_2_ electrode without the presence of any added salts was 73.8 mV/pH. In the presence of 0.01M of KCl, CaCl_2_, MgCl_2_, Na_2_SO_3_, and NH_4_NO_3_, the pH sensitivity in the range of 2–12 was 73.4 mV/pH, 73.1 mV/pH, 71.9 mV/pH, 69.8 mV/pH, and 73.8 mV/pH, respectively. Therefore, we observed small advantageous changes in comparison to the pH sensitivity exhibited by the pH electrodes in the absence of any additional ions, which reflects good selectivity of the fabricated electrodes. A similar result was observed for pure RuO_2_-based pH electrodes [6] in the presence of KCl and NH_4_NO_3_, which indicates that the incorporation of Co_3_O_4_ in the pH-sensing layer does not significantly impact the selectivity of the final screen-printed layer. The presence of the ions investigated in this work also had no significant influence on the selectivity of La_2_O_3_-RuO_2_-based pH-sensing electrodes [13]. The largest influence of the interference effect was observed in the presence of Na_2_SO_3_, whereas the least influence was related to NH_4_NO_3_. On the other hand, the highest influence on pH sensitivity was observed in the presence of NH_4_NO_3_ in a screen-printed layer based on 50 mol% CuO-50 mol% RuO_2_ and covered with a proton-selective Nafion membrane [14].

### 3.3. Application in Real-Life Water Samples

In water quality monitoring, the quality of the water data is as important as the water data itself. Accuracy and precision are critical for well-calculated and effective decision making, which is especially very important when public health, environmental impact, and financial repercussions are taken into consideration.

Since safe water guidelines are defined with a precision of approximately two decimal points, it is essential for fabricated sensors to achieve a comparable level of accuracy. Tested samples include municipal water from the tap, commercial water from packaged mineral water bottles, environmental water samples from the surface of Vistula River and Zakrzówek Lake in Kraków, Poland. Additionally, water samples from the surface and depths of 2 m, 42 m, 75 m, and 100 m of the Baltic Sea were also tested. All the developed pH-sensing layers based on Co_3_O_4_ and RuO_2_ demonstrated a reasonable level of accuracy compared to a glass electrode, with a low standard deviation (Table 3). However, the most accurate composition across all water samples was 50 mol% Co_3_O_4_—50 mol% RuO_2_. The observed accuracy of the pH electrodes, in contrast to that of the glass electrode, was comparable to the accuracy observed for pure RuO_2_-based pH electrodes in real-life water samples [6]. With regard to the interference effect, the ions were found in all types of water, whether it was mineral water for drinking or groundwater for agriculture. It is important for pH sensors to selectively measure the concentration of protons in the solution because if other ions are also measured, it no longer measures the acidity or alkalinity but instead reflects the total conductivity of the solution which includes many other ions that are present in various samples of water, especially the contaminated and polluted ones. The true measurement of pH also helps to take correct action in terms of chemical remediation of polluted waters. Incorrect pH measurement and consequently developed wrong solutions may further deteriorate the water environment and contradictorily damage its quality rather than improve it. Some reactions are irreversible, and therefore, the decision to implement them should be based on good quality and dependable data from accurate sensors which account for phenomena such as drift effects, interference effects, and the impact of conductivity and temperature on the Nernst equation and sensitivity characteristics of the sensors.

Although very minor, the disparity between the sensors was more pronounced in samples of tap, river, and lake water. The water from the sea had much higher conductivity in comparison. The average conductivity of the Baltic Sea samples was 16.9 mS/cm, whereas the average conductivity of the tap, river, and lake water was 2.4 mS/cm. It was shown previously that the electrodes based on the combination of Co_2_O_3_ and ruthenium oxide have better stability in samples with higher conductivity [16].

The pH in the water from Gdańsk Deep showed a consistent decrease with depth from the sea surface to 100 m (Table 3). This vertical gradient is in line with spring pH vertical distribution patterns observed in open waters of the Baltic Sea waters [29,30]. The dynamics of surface pH is primarily controlled by the balance between primary production and respiration. During spring blooms, photosynthetic CO_2_ assimilation temporarily raises the surface pH. In deeper waters, the decomposition of organic matter releases CO_2_, what leads to decrease of pH. Stratification, marked by a permanent halocline at depths of approximately 60–80 m, intensifies these processes by limiting vertical mixing between the surface and bottom layers, allowing CO_2_ to accumulate in bottom waters. The Baltic Sea’s naturally low buffering capacity amplifies pH variations, while additional factors such as eutrophication and oxygen depletion in hypoxic zones introduce additional complexity to these chemical dynamics [30]. Table 3 also presents the salinity values determined for the Baltic Sea water on various depths. These values are very close to the typical range of salinity for the Baltic Sea Proper (this part of the Baltic Sea), which is usually ~7.28 PSU in the surface and 12–13 PSU in the bottom. Thus, the observed trend of the pH decrease with increasing depth is also related to enhanced salinity.

Direct pH measurements in Baltic waters remain limited. Typically, acid-base properties are assessed through indirect calculations using total alkalinity (TA), dissolved inorganic carbon (DIC), or pCO_2_ measurements [31,32], primarily focusing on surface waters [33,34]. Challenges in direct pH measurement arise from limitations in analytical techniques, complexities in data interpretation, and difficulties in accurately parameterizing acid-base processes for numerical modeling [32].

Notable advancements include the adaptation of spectrophotometric pH measurements with m-cresol purple for the Baltic Sea conditions [35]. Future analytical improvements may be aided by the development of pH reference materials for brackish water [32]. Additionally, technologies such as solid-state pH sensors show promising potential. This is especially relevant in marine environments, as the widely used ISFET sensors, which once served as an alternative to spectrophotometric pH analyses, are no longer in production.

The ongoing scarcity of comprehensive acid-base property data, particularly direct pH measurements, highlights the critical need for the development and implementation of reliable and accessible pH-monitoring systems, given their importance for marine and climate studies.

### 3.4. Anti-Bacterial Properties of the Electrodes

**Figure 8:**
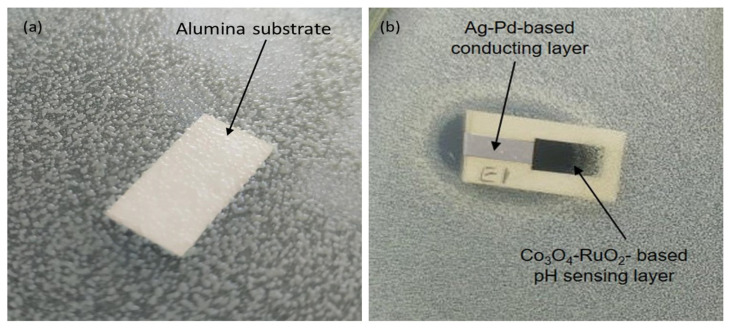
Comparison of the inhibition of the growth of bacterial colony around an alumina substrate (**a**) and a Ag-Pd-based conducting layer and Co_3_O_4_-RuO_2_-based pH-sensing layer (**b**).

The results of the preliminary test to investigate the influence of the developed sensing layer on bacterial growth are shown in Figure 8. The antibacterial effect was observed in the form of inhibition of colony growth. The ceramic substrate based on alumina (96% Al_2_O_3_) without any metallic or metal oxide layers was microbiologically neutral, showing no bacterial growth inhibition (Figure 8a). A strong growth inhibition was observed over a large diffusion zone surrounding the entire component containing a Ag-Pd conducting layer, suggesting a significant antibacterial effect against *Escherichia coli* (Figure 8b). For layers deposited with a Co_3_O_4_-RuO_2_-based thick-film paste, reduced inhibition of colony growth was observed, evidenced by a shorter distance between the colonies and the sample compared to the layers based on a Ag-Pd conducting layer. These findings confirm that the observed bacterial growth inhibition is primarily due to the presence of the Ag-Pd layer rather than the 50 mol% Co_3_O_4_-50 mol% RuO_2_-based pH-sensing electrode. This observation suggests the potential for further research to explore the compound’s influence on microbial activity; however, preliminary studies have already identified the sensing layer compatibility with the bacterial environment (Figure 8b).

Maintaining minimal interaction between the sensor layer and microorganisms is crucial for operation in biological environments, as it preserves the natural state of microbial colonies and ensures stable, long-term measurements and reliable data. However, inhibiting bacterial growth near the electrode can affect local biochemical stability by altering parameters such as pH and redox potential through microbial metabolism. Bacterial metabolism significantly influences the pH of the surrounding environment. This arises from the production or consumption of protons during metabolic activities, which can lead to acidification or alkalization [36,37]. Different bacterial species exhibit distinct proton-dynamics behaviors. For example, *Escherichia coli* consume protons during aerobic growth with acetate, whereas Geobacter species produce protons when oxidizing acetate with Fe (III) as the electron acceptor. The presence of bacteria can alter the pH of their surrounding environment due to the accumulation of acid anions within cells, regulation of internal pH or changes in the composition of bacterial cell membranes [38,39]. Reported examples include *Escherichia coli* and *Listeria monocytogenes*, which produce organic acids like lactic and acetic acids, thereby lowering the pH of their environment [39].

Considering the above, the neutral nature of the sensor components and minimization of their impact on the environmental activity are key considerations in designing sensors that need to operate in close proximity to living organisms. In the fabricated electrode, only the sensitive layer was exposed to the measuring environment, and therefore, the silver–palladium-based conducting layer did not come in contact with the water. This indicates that the pH measurements made using the fabricated electrodes in real-life environmental water samples was not altered by the anti-bacterial properties of the conducting layer. However, further research of alternative materials that do not disrupt the bio-chemical stability of their immediate environments could aid in minimizing the negative impact of electrodes and other electronic components during their end-of-life stage of the life cycle.

## 4. Conclusions

This study demonstrated that reducing the RuO_2_ content in pH-sensing layers by incorporating Co_3_O_4_ is feasible across a wide range of molar proportions in the starting metal oxide powders in the screen-printing process. The pH electrode based on 50 mol% RuO_2_—50 mol% Co_3_O_4_ exhibited the best balance of performance and stability. While higher Co_3_O_4_ content led to issues such as increased drift, slower response time, and poor adhesion, the optimized composition based on 50 mol% RuO_2_—50 mol% Co_3_O_4_ maintained excellent potentiometric characteristics, sensitivity to pH changes, selectivity, and stability across varying temperatures (0–60 °C) and salinity levels (7–12 PSU). Morphological, structural, and elemental properties were investigated in this study for a deeper understanding of the differences between each type of pH-sensing layer. XRD analysis confirmed the formation of a nonstoichiometric ruthenium cobalt spinel oxide, and the sensors were accurate when tested against commercial glass electrodes in environmental water samples, including those from various depths of the Baltic Sea. This work highlights a promising approach to reducing reliance on rare and expensive RuO_2_ by combining it with the more abundant and comparatively cheap Co_3_O_4_ in screen-printed pH-sensing electrodes while maintaining high-performance pH-sensing capabilities.

## Figures and Tables

**Table 1:** Roughness profile parameters for 50 mol% Co_3_O_4_-50 mol% RuO_2_.

Maximum peak height R_p_ (μm)	3.217
Maximum valley depth R_v_ (μm)	3.871
Maximum height R_z_ (μm)	7.088
Average height R_c_ (μm)	3.541
Arithmetic average of height deviations R_a_ (μm)	1.101
Asymmetry R_sk_	−0.2143
Kurtosis R_ku_	2.784

**Table 2:** Potentiometric characteristics of the electrodes with different proportions of Co_3_O_4_ and RuO_2_.

mol% Co_3_O_4_	mol% RuO_2_	Sensitivity (mV/pH)	R^2^	*E*^0^ (mV)
0	100	61.8 ± 1.0	0.996	681.9 ± 5.00
30	70	53.1 ± 9.5	0.998	672.1 ± 57.5
40	60	69.5 ± 1.3	0.999	841.1 ± 50.6
50	50	74.6 ± 1.1	0.998	891.5 ± 20.0
60	40	64.9 ± 6.1	0.999	814.3 ± 43.7
70	30	65.8 ± 0.4	0.999	780.7 ± 35.5
100	0	42.8 ± 16.8	0.982	575.3 ± 52.9

**Table 3:** The pH values of real-life water samples measured using a glass electrode and the electrodes with different proportions of Co_3_O_4_ and RuO_2_.

Water Sample	Average Salinity (PSU *)	pH (Glass Electrode)	pH Measured with Co_3_O_4_-RuO_2_ Electrodes				
			mol% of Co_3_O_4_ in the Sensing Layer				
			30	40	50	60	70
Tap	-	7.94	7.90 ± 0.08	7.91 ± 0.04	7.93 ± 0.01	7.78 ± 0.04	7.63 ± 0.01
Mineral	-	5.45	5.38 ± 0.01	5.46 ± 0.00	5.44 ± 0.01	5.44 ± 0.01	5.41 ± 0.04
River	-	8.10	8.01 ± 0.07	8.10 ± 0.00	8.10 ± 0.02	7.89 ± 0.09	8.09 ± 0.01
Lake	-	8.18	8.12 ± 0.03	8.22 ± 0.02	8.15 ± 0.00	8.03 ±0.05	7.85 ± 0.01
Sea (surface)	7.26	8.28	8.23 ± 0.07	8.24 ± 0.04	8.27 ± 0.03	8.24 ± 0.04	8.20 ± 0.10
Sea (2 m deep)	7.26	8.22	8.19 ± 0.03	8.20 ± 0.06	8.23 ± 0.01	8.18 ± 0.07	8.15 ± 0.01
Sea (42 m deep)	7.48	7.95	7.91 ± 0.08	7.93 ± 0.02	7.94 ± 0.01	7.89 ± 0.06	7.87 ± 0.02
Sea (72 m deep)	10.23	7.49	7.53 ± 0.05	7.51 ± 0.03	7.49 ± 0.02	7.55 ± 0.05	7.55 ± 0.10
Sea (100 m deep)	12.11	7.45	7.48 ± 0.04	7.49 ± 0.25	7.46 ± 0.01	7.48 ± 0.04	7.50 ± 0.01

* Practical salinity unit (1 g of salt per 1000 g of water).

## Data Availability

Data are available upon request from the authors.
